# Cataract Surgery and Mental Health: A Comprehensive Review on Outcomes in the Elderly

**DOI:** 10.7759/cureus.65469

**Published:** 2024-07-26

**Authors:** Kasturi K Dhawale, Pravin Tidake

**Affiliations:** 1 Ophthalmology, Jawaharlal Nehru Medical College, Datta Meghe Institute of Higher Education and Research, Wardha, IND

**Keywords:** quality of life, cognitive function, anxiety, depression, elderly mental health, cataract surgery

## Abstract

Cataract surgery is a widely performed and highly effective procedure that significantly improves vision in elderly patients. This narrative review examines the impact of cataract surgery on mental health outcomes in the elderly, focusing on conditions such as depression, anxiety, and cognitive decline. The review highlights the prevalence of cataracts in older adults and the importance of mental health in this demographic, emphasizing the interconnectedness of visual and mental health. Improved vision following cataract surgery has been associated with enhanced quality of life, increased independence, and better psychological well-being. Mechanisms linking visual improvement to mental health benefits include biological pathways, psychosocial factors, and overall health improvements. However, socioeconomic factors, access to healthcare, and patient education play crucial roles in achieving positive outcomes. This review also compares cataract surgery with other interventions, providing a cost-benefit analysis and discussing the long-term sustainability of mental health benefits. Practice recommendations include pre-surgical mental health screening, integrative care approaches, and guidelines for postoperative care focusing on mental health. The review concludes with suggestions for future research to further explore the relationship between cataract surgery and mental health in the elderly, aiming to enhance clinical practice and public health strategies.

## Introduction and background

Cataract surgery is a common and highly effective procedure to replace the eye's cloudy lens with an artificial intraocular lens (IOL) [1]. This surgery is typically performed on an outpatient basis and boasts a high success rate, with most patients experiencing significant improvements in vision. There are two main types of cataract surgery: phacoemulsification and extracapsular cataract extraction (ECCE). Phacoemulsification involves emulsifying the lens with an ultrasonic handpiece and aspirating it from the eye, while ECCE entails removing the lens in one piece through a larger incision. Advanced surgical techniques and technology have made cataract surgery one of the safest and most routinely performed worldwide [2]. Cataracts are a leading cause of visual impairment globally, particularly affecting the elderly population. As individuals age, the proteins in the eye’s lens can clump together, forming a cataract and cloudy vision. According to the World Health Organization, cataracts contribute to approximately 51% of world blindness, affecting millions [3]. In developed countries, cataract surgery is readily available and widely performed. In contrast, in developing regions, access to this surgery can be limited, leading to higher rates of visual impairment and blindness among the elderly [4].

Mental health is critical to overall well-being, particularly in older adults. As people age, they may face numerous challenges that can impact their mental health, including physical health issues, loss of loved ones, and changes in social roles and support systems [5]. Common mental health conditions in the elderly include depression, anxiety, and cognitive decline, which can significantly affect their quality of life. Vision impairment due to cataracts can exacerbate these issues, leading to increased social isolation, decreased independence, and overall poorer mental health outcomes. Therefore, addressing both the visual and mental health needs of the elderly is essential for promoting their overall well-being and quality of life [6]. This narrative review aims to explore the relationship between cataract surgery and mental health outcomes in the elderly. The specific objectives are to examine the impact of cataract surgery on visual and functional outcomes in older adults and investigate the effects of improved vision post-surgery on mental health conditions such as depression, anxiety, and cognitive function. Additionally, the review aims to identify the mechanisms linking visual improvement to better mental health, discuss the barriers and facilitators to achieving positive mental health outcomes following cataract surgery, provide recommendations for integrating mental health support in the preoperative and postoperative care of elderly cataract patients, and highlight areas for future research to understand further the interplay between vision improvement and mental health in the elderly population.

## Review

Visual and functional outcomes of cataract surgery

Improvement in Visual Acuity (VA)

Cataract surgery has been demonstrated to enhance VA significantly in elderly patients. Multiple studies have reported substantial improvements in VA following the procedure. One study concluded that cataract surgery effectively alleviates depressive symptoms in the elderly, with improved VA contributing to reduced cognitive impairment in older patients. Another study found that the mean VA improvement after phacoemulsification cataract surgery was 0.33 (95% CI: 0.31-0.35), increasing from a mean best-corrected VA of 0.54 preoperatively to 0.87 postoperatively [7]. A Singapore study reported that 85.1% of patients experienced improved acuity after cataract surgery. An Auckland study of 488 eyes revealed that 88% of eyes achieved a VA of 6/12 or better postoperatively. As expected, VA improved significantly after cataract surgery in the Collaborative Initial Glaucoma Treatment Study (CIGTS), with a mean improvement of over four lines of vision (21.6 letters on the Early Treatment of Diabetic Retinopathy Study (ETDRS) VA chart; p < 0.0001) [8]. However, the quality of visual outcomes after cataract surgery varies widely across different countries and regions. A systematic review found that the proportions of participants with postoperative presenting VA ≥ 0.32 (20/60) ranged from 29.9% to 80.5% in studies from low- and middle-income countries [9].

Enhancement of Daily Functioning and Quality of Life

Cataract surgery has significantly enhanced daily functioning and quality of life in elderly patients. Multiple studies have demonstrated that cataract surgery improves visual function, cognitive function, emotional well-being, and overall quality of life. The Sunderland Cataract Study revealed that surgery significantly improves visual function and cognitive, emotional, and general well-being. Improved physical function, a critical outcome of cataract surgery, includes an increased ability to perform activities of daily living and relief from the fear of blindness. Studies have found that regardless of preoperative VA in the better eye, most patients reported improved ability to perform visually dependent tasks after cataract surgery [10]. Improved visual function following cataract surgery can mitigate the progressive deterioration of quality of life observed in elderly patients. Cataract extraction improved visual function and health-related quality of life in 464 patients aged 65. After cataract surgery, patients often notice a significant improvement in their overall quality of life. Their newly clear eyesight enables them to enjoy hobbies and activities they may have had to forgo due to poor vision caused by cataracts [11]. Restoring vision through cataract surgery can boost self-esteem, stabilize moods, and leave patients feeling more confident and satisfied in their daily lives. Cataract surgery is highly likely to enhance daily functioning and quality of life in older adults by improving visual function, cognitive function, emotional well-being, and the ability to perform activities of daily living. The benefits range from improved physical mobility to better mental health and overall quality of life [12].

Comparison of Different Surgical Techniques and Their Outcomes

Cataract surgery has significantly improved visual and functional outcomes in elderly patients. Numerous studies have found that cataract surgery leads to improvements in VA, depression, anxiety, and cognitive function in older adults. One study concluded that cataract surgery is effective in alleviating depressive symptoms in the elderly, with improved VA having far-reaching effects on reducing cognitive impairment in older patients. Another study demonstrated that cataract surgery did not predispose patients to significant cognitive deterioration in the perioperative period or during the first postoperative year [6]. However, the quality of visual outcomes after cataract surgery varies widely across different countries and regions. A systematic review found that the proportions of participants with postoperative presenting VA ≥ 0.32 (20/60) were consistently over 70% in high-income country studies but mostly below 70% in low- and middle-income country studies, ranging from 29.9% to 80.5%. Significant differences in postoperative VA were also observed within individual countries. The leading causes of postoperative visual impairment (defined mostly as presenting VA < 20/60) included refractive error, ocular comorbidities, and surgical complications such as posterior capsule opacification. One study in Nigeria identified aphakia as the leading cause [9].

Several studies compare different surgical techniques and their outcomes across various medical specialties. A worldwide multicenter study compared outcomes of robot-assisted minimally invasive gastrectomy (RAMIG) techniques. Key findings include high lymph node yield (median 31-34 nodes), with ≥15 nodes retrieved in 92-96% of cases, high radicality rates of 93-96%, acceptable overall complication rates of 23-42%, low anastomotic leakage rates of 2-10%, and 30-day mortality of 1%. These outcomes were comparable to or better than results from multicenter trials and population-based studies of open and laparoscopic gastrectomy [13].

A study compared outcomes of surgical thrombectomy vs. de novo arteriovenous fistula (AVF) creation for treating acute AVF thrombosis. Technical success was 83% for thrombectomy, with one-month patency rates of 80% for thrombectomy vs. 100% for de novo AVF and six-month patency rates of 25% for thrombectomy vs. 91% for de novo AVF, a statistically significant difference. Another study compared open radical retropubic prostatectomy (RRP) to laparoscopic radical prostatectomy (LRP) for prostate cancer. Positive surgical margin rates were similar between RRP (28.6%) and LRP (26.5%), but for organ-confined disease, positive margins were higher with LRP (17.5%) vs. RRP (10.5%, p = 0.006). It remains unclear if this higher positive margin rate with LRP leads to higher biochemical recurrence. A review article discussed three main surgical approaches for primary infected aortic aneurysms: open in-situ repair, extra-anatomic repair, and endovascular aneurysm repair (EVAR). However, no specific outcomes data comparing these techniques was provided [14].

Mental health outcomes post-cataract surgery

Impact on Depression and Anxiety Levels

Cataract surgery has been shown to reduce symptoms of depression and anxiety in older adults significantly. Several studies have reported notable improvements in mental health outcomes following the procedure. A population-based study using linked data found that first eye cataract surgery reduced mental health contacts for depression and anxiety by 18.8% in the year following the surgery [15]. Additionally, a nationwide cohort study demonstrated that cataract surgery was associated with a 25% decreased risk of depression compared to those who did not undergo the surgery [6]. A comprehensive review highlighted a notable correlation between cataract surgery and reduced anxiety and depression, suggesting its potential in alleviating these mental health concerns [6]. A multisite longitudinal study also found that successful cataract surgery resulted in a significant drop in both depressive and generalized anxiety symptoms [16]. However, the effectiveness of cataract surgery in addressing mental disorders with multifaceted causative factors may vary. Significant predictors of depression at the end of the study year included objective vision changes, subjective adjustment to the effects of surgery, and high depression scores before surgery [6]. Moreover, preoperative anxiety and depression significantly impact cataract surgery outcomes [17]. While cataract surgery appears highly likely to improve mental health in older adults, primarily in visual impairment, the scope of improvement may not fully encompass the broader spectrum of psychological well-being, which is influenced by multifaceted factors beyond VA alone. Future studies are required to develop a multifactor strategy for the prevention and management of depression in patients with cataracts or visual impairment.

Changes in Cognitive Function

**Figure 1:**
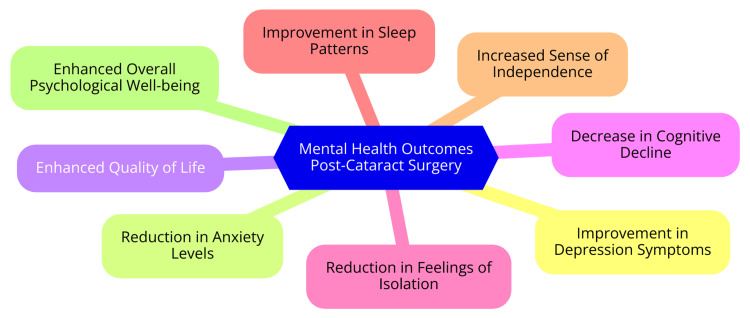
Mental health outcomes post-cataract surgery Image credit: Dr. Kasturi K. Dhawale

Several studies have found that cataract surgery can improve cognitive function in elderly patients. A prospective observational study in Japan found that cataract surgery significantly increased cognitive test scores in older patients with mild cognitive impairment (MCI). The MCI group showed significant improvement in Mini-Mental State Examination (MMSE) scores from before to after surgery (25.65 ± 1.03 vs. 27.08 ± 1.99, p < 0.001). Cognitive function improved significantly more in the MCI group than in the dementia group after surgery. The study concluded that the likelihood of cognitive improvement after cataract surgery highly depends on a patient's preoperative cognitive state [18]. A study in England found that one year after cataract surgery, participants with normal cognition had significantly better visual outcomes than those with impaired cognition. Regression analyses showed a relationship between baseline cognition and VA at one year. This suggests that while patients with impaired cognition benefit from cataract surgery, they do not benefit to the same extent as patients with normal cognition [19]. A 13-year longitudinal study using a nationally representative sample found that cataract surgery was associated with a reduction in the rate of cognitive decline over time. The rate of cognitive decline among individuals with cataracts became gentler after surgery and similar to that of individuals without cataracts. This study supported the hypothesis that cataract-induced visual deprivation may accelerate cognitive decline, which can be slowed by cataract surgery [20]. A study of 108 subjects over 60 years old undergoing cataract surgery found that the surgery did not predispose them to significant cognitive deterioration in the perioperative or during the first postoperative year. However, objective vision changes, subjective adjustment to surgery effects, and high depression scores before surgery were significant predictors of depression at the end of the study year [21]. While cataract surgery appears to have a positive impact on cognitive function in many older adults, the extent of improvement may depend on factors such as preoperative cognitive state, depression, and subjective adjustment to surgery. Cataract-induced visual deprivation may accelerate cognitive decline, which can potentially be slowed by restoring vision through cataract surgery. Mental health outcomes post-cataract surgery are shown in Figure 1.

Mechanisms linking cataract surgery and mental health

Biological and Neurological Pathways

Several biological and neurological pathways have been proposed to explain the link between cataract surgery and improved mental health outcomes in older adults. Cataract surgery restores visual input to the brain, potentially stimulating cognitive abilities and slowing age-related cognitive decline. Improved VA can enhance engagement in daily activities, social interactions, and hobbies, providing mental stimulation and reducing the risk of cognitive impairment [17]. The restoration of visual input after cataract surgery may trigger neuroplastic changes in the brain, allowing for the reorganization of neural networks involved in visual processing and cognitive functions. This brain plasticity may contribute to improved cognitive performance and a reduced risk of dementia. Additionally, cataract surgery may reduce oxidative stress and inflammation in the brain, both of which are associated with cognitive decline and mental health disorders. Removing the cataract and the subsequent improvement in visual function may decrease inflammation and oxidative stress, potentially leading to better cognitive and mental health outcomes [22]. Vision impairment due to cataracts can disrupt circadian rhythms and sleep patterns, which are closely linked to mental health. Cataract surgery may restore normal sleep patterns, improving sleep quality and reducing the risk of depression and anxiety. Furthermore, vision impairment increases the risk of falls and related injuries in older adults, which can have negative psychological consequences. Cataract surgery has been shown to reduce the incidence of falls, potentially improving confidence, independence, and overall mental well-being [23].

Psychosocial Factors

Cataract surgery has been shown to positively impact mental health outcomes in older adults, with several studies demonstrating improvements in depression, anxiety, and cognitive function. The mechanisms linking cataract surgery to mental health are multifaceted, involving both biological and neurological pathways. Improved VA after surgery can stimulate cognitive abilities, trigger neuroplastic changes in the brain, and reduce oxidative stress and inflammation. Additionally, cataract surgery may restore normal sleep patterns, reduce the risk of falls and injuries, and improve overall quality of life [17]. Psychosocial factors also play a significant role in the relationship between cataract surgery and mental health. Enhanced vision can increase social engagement, enabling older adults to participate more easily in daily activities and social interactions. This increased engagement provides mental stimulation, reduces feelings of isolation and loneliness, and improves overall well-being. Cataract surgery can also enhance independence by improving functional ability and reducing the risk of falls, which boosts confidence and self-esteem. Furthermore, improved vision may reduce the burden on caregivers, leading to an improved quality of life for both patients and their family members [17]. However, it is important to recognize that the effectiveness of cataract surgery in addressing mental disorders with multifaceted causes may vary. Factors, such as genetics, physical illnesses, and life events, can also contribute to anxiety and depression in older adults. Therefore, a comprehensive approach addressing visual impairment and broader psychosocial factors is essential for promoting mental well-being in this population. Future research should focus on developing multifactor strategies for the prevention and management of depression in patients with cataracts or visual impairments, taking into account the complex interplay between biological, neurological, and psychosocial factors [24].

Reduction in Comorbidities and Overall Health Improvement

Cataract surgery can reduce certain comorbidities and improve overall health in elderly patients. One significant benefit is the reduced risk of falls and injuries. Vision impairment due to cataracts increases the risk of falls and related injuries in older adults. Cataract surgery has been shown to reduce the incidence of falls, thereby improving confidence, independence, and overall well-being [25]. Another potential benefit is improved cognitive function. Some studies suggest that cataract surgery may reduce the risk of developing dementia or cognitive impairment. The restoration of visual input may stimulate cognitive abilities and slow down age-related cognitive decline. This is particularly important for maintaining mental sharpness and quality of life in older adults [26]. Cataract surgery can also enhance social engagement. Improved vision after surgery can encourage older adults to participate in social activities, reducing feelings of isolation and loneliness. These factors are known risk factors for depression and anxiety, which can further impact overall health and well-being [17]. Several studies have found that cataract surgery can lead to improvements in depression and anxiety in elderly patients. Improved VA after surgery was found to have far-reaching effects on reducing cognitive impairment in older patients. Reducing mental health comorbidities can contribute to a better quality of life and overall health [25].

Barriers and facilitators to positive outcomes

Socioeconomic Factors

Socioeconomic factors play a significant role in influencing cataract surgery outcomes and uptake, particularly among elderly patients. Lower education and household income levels are associated with a higher risk of cataracts and a lower likelihood of undergoing cataract surgery, especially in women. This disparity is likely due to reduced access to healthcare and awareness of treatment options among lower socioeconomic groups [27]. Lack of health insurance or inadequate coverage for cataract surgery is another major barrier, leading to significant socioeconomic disparities in access to treatment. Patients without insurance often cannot afford the out-of-pocket costs, preventing them from undergoing the procedure. The high cost of cataract surgery is a crucial factor preventing many patients, especially those from low-income backgrounds, from seeking treatment. Providing free or subsidized surgical camps can help overcome this barrier and improve access to care [28]. Poor awareness about cataracts and the availability of treatment options is more common among those with lower education and socioeconomic status. Elderly patients, especially those over 66, may have difficulty traveling to surgical locations without assistance. Lack of family support and escort can prevent them from accessing care. Locating surgical camps close to patients' homes and providing transportation assistance can facilitate uptake and improve outcomes [29].

Access to Healthcare and Surgical Services

Several studies have identified key barriers and facilitators to accessing healthcare and surgical services for the elderly, especially in rural areas. Financial constraints are a significant reason elderly patients do not access services, as lack of financial security and high healthcare costs prevent them from seeking care. Subsidized surgical camps and health insurance can help address this barrier [30]. Another significant issue is the lack of awareness among elderly patients. Many need to be made aware of the severity of their condition or that treatment options are available. Community engagement and education are essential to inform and encourage elderly patients to seek care. Lack of family support is also a challenge, as elderly patients, especially those over 66, often have difficulty traveling to healthcare facilities without assistance from family members [31]. Geographic barriers pose a significant obstacle, as the unavailability of accessible healthcare facilities, especially in rural areas, makes it difficult for elderly patients to access services. Psychological factors, such as fear of surgical complications and the perception that an ailment is not severe enough, can also prevent elderly patients from seeking care [30]. Proximity of services is a crucial facilitator, as providing healthcare and surgical camps close to elderly patients' homes significantly improves access. Affordability is another crucial factor, with free or subsidized services, including health insurance coverage, enabling more elderly patients to utilize care. Community engagement, involving community health workers and previously treated patients, helps provide information and encouragement to elderly patients [32].

Patient Education and Awareness

Lack of awareness about cataracts and treatment options is a significant barrier to accessing care. Many patients may need to realize the severity of their condition or that treatment is available. Effective methods to provide information and raise awareness include announcements, pamphlets, and community health workers. According to one study, 57.3% of patients reported these as the most common sources of information on cataract surgery [29]. Misconceptions and fears can also prevent patients from seeking timely treatment. Some believe they should wait until the cataract is fully mature before having surgery. Educating patients about the benefits and safety of cataract surgery is crucial. Statistics show that nine out of 10 people see better after surgery, with colors often brighter due to the clear artificial lens. Detailed information on what to expect before, during, and after surgery helps prepare patients. This includes instructions on fasting, medications, activity restrictions, and follow-up care [33]. Involving community health workers and previously operated patients in outreach efforts can motivate more people to undergo surgery by providing information, support, and encouragement. Comprehensive patient education and awareness campaigns are crucial to address knowledge gaps, allay fears, and encourage the timely uptake of cataract surgery, especially in underserved communities. A combination of informational materials, community engagement, and patient testimonials can be effective strategies [34].

Support Systems and Post-surgical Care

Post-surgical care and support systems are crucial to recovery after cataract surgery. A supportive network of family and friends to assist with transportation, daily tasks, and emotional support is invaluable during recovery. Community organizations, such as local senior centers, churches, or non-profits, may offer transportation assistance or other support services for elderly patients. Additionally, connecting with others who have undergone cataract surgery through online forums can provide encouragement and practical advice [35]. Patients must carefully follow their ophthalmologist's instructions for post-surgical care. This includes proper medication management, such as eye drops, to prevent infection and reduce inflammation. Activity restrictions, such as avoiding strenuous activities, bending over, and rubbing the eye, are important for proper healing. Regular check-ups with the surgeon are essential to monitor healing and address any complications. A nutrient-rich diet with plenty of hydration can support the healing process, with foods high in vitamins C and E, omega-3s, and antioxidants particularly beneficial [36]. While complications are rare, patients need to be aware of potential issues that may arise. Symptoms like severe pain, redness, and vision loss may indicate an infection and require immediate medical attention. Mild swelling and discomfort are common but should subside within a few days. In rare cases, a sudden increase in floaters or flashes of light may indicate retinal detachment, which warrants prompt evaluation [37].

Comparative Analysis: Cataract Surgery vs. Other Interventions

Cataract surgery is one of the most effective and cost-efficient interventions compared to ophthalmologic and non-ophthalmologic treatments. A study found that every dollar invested in cataract treatment returns $20.50 on average, more than double the return on investment compared to programs aimed at cardiovascular disease, adolescent health, and maternal and child health. This finding highlights the significant cost-effectiveness of cataract surgery and its potential for providing substantial economic benefits [38]. When comparing cataract surgery techniques, phacoemulsification, the most common method, offers better visual outcomes than ECCE in terms of uncorrected VA. Manual small incision cataract surgery (SICS) provides better visual outcomes than ECCE but slightly inferior unaided VA compared to phacoemulsification. These findings suggest that phacoemulsification may be the most effective surgical technique for improving visual outcomes in cataract patients [39].

Cataract surgery has a strong potential to enhance mental well-being, particularly concerning issues related to visual impairment. However, the effectiveness of cataract surgery in addressing mental disorders with multifaceted causative factors may vary. Preoperative anxiety and depression significantly impact cataract surgery outcomes, highlighting the importance of considering the patient's mental health status before surgery and recognizing the potential limitations of cataract surgery in addressing complex psychological factors [17]. Cataract surgery is highly cost-effective compared to other interventions and provides superior visual outcomes to alternative ophthalmologic treatments. While cataract surgery can improve mental health, particularly related to visual impairment, the long-term sustainability of these benefits may be influenced by preoperative mental health status and the complexity of underlying psychological factors. These findings underscore the importance of a comprehensive approach to patient care, considering both the physical and mental health implications of cataract surgery [40].

Future research directions

Future research directions in cataract surgery and mental health outcomes in the elderly should focus on identifying gaps in current research, conducting longitudinal studies, and exploring different demographic and geographic populations. Most studies have focused on the short-term effects of cataract surgery on mental health. More research is needed to understand the benefits' long-term impact and durability. Additionally, most studies have been conducted in developed countries; more research is needed in developing countries to understand the impact of cataract surgery on mental health in different socioeconomic and cultural contexts. While most studies have focused on the impact of cataract surgery on depression and anxiety, more research is needed to understand the impact on other mental health outcomes, such as cognitive function and quality of life [20]. Longitudinal studies that follow patients for several years after cataract surgery would provide valuable insights into the long-term impact of the surgery on mental health outcomes. These studies could also help identify factors that predict which patients are most likely to benefit from cataract surgery in terms of mental health outcomes. Furthermore, longitudinal studies could help understand the impact of cataract surgery on the progression of cognitive decline and the development of dementia. More research is needed to understand the impact of cataract surgery on mental health outcomes in different age groups such as younger and middle-aged adults. Research is also needed to understand the impact of cataract surgery on mental health outcomes in different ethnic and racial groups, as well as in different socioeconomic groups. Comparative studies across different geographic regions and healthcare systems could provide valuable insights into the impact of cultural and healthcare system factors on the relationship between cataract surgery and mental health outcomes [40]. Future research in the field of cataract surgery and mental health outcomes in the elderly should focus on identifying gaps in current research, conducting longitudinal studies, and exploring different demographic and geographic populations. This research could provide valuable insights into the long-term impact of cataract surgery on mental health outcomes and help inform clinical practice and healthcare policy.

## Conclusions

Cataract surgery, a highly effective procedure for restoring vision, plays a crucial role in enhancing the quality of life for elderly individuals. Beyond the obvious visual benefits, this surgery significantly impacts mental health outcomes, including reductions in depression, anxiety, and cognitive decline. Improved vision leads to greater independence, increased social interaction, and overall enhanced psychological well-being. Despite these benefits, various barriers, such as socioeconomic factors and limited access to healthcare services, can hinder the positive outcomes of cataract surgery. Therefore, integrating mental health support into the preoperative and postoperative care of cataract patients is essential. Future research should focus on understanding the long-term mental health benefits of cataract surgery and exploring strategies to make this life-changing procedure accessible to all elderly individuals, regardless of their socioeconomic status. This comprehensive approach will ensure that the elderly not only regain their vision but also experience an improved quality of life through better mental health.
